# Selective Genomic Copy Number Imbalances and Probability of Recurrence in Early-Stage Breast Cancer

**DOI:** 10.1371/journal.pone.0023543

**Published:** 2011-08-12

**Authors:** Patricia A. Thompson, Abenaa M. Brewster, Do Kim-Anh, Veerabhadran Baladandayuthapani, Bradley M. Broom, Mary E. Edgerton, Karin M. Hahn, James L. Murray, Aysegul Sahin, Spyros Tsavachidis, Yuker Wang, Li Zhang, Gabriel N. Hortobagyi, Gordon B. Mills, Melissa L. Bondy

**Affiliations:** 1 Department of Cellular and Molecular Medicine, Arizona Cancer Center, University of Arizona, Tucson, Arizona, United States of America; 2 Department of Clinical Cancer Prevention, MD Anderson Cancer Center, University of Texas, Houston, Texas, United States of America; 3 Department of Biostatistics, MD Anderson Cancer Center, University of Texas, Houston, Texas, United States of America; 4 Department of Bioinformatics and Computational Biology, MD Anderson Cancer Center, University of Texas, Houston, Texas, United States of America; 5 Department of Pathology, MD Anderson Cancer Center, University of Texas, Houston, Texas, United States of America; 6 Department of Breast Medical Oncology, MD Anderson Cancer Center, University of Texas, Houston, Texas, United States of America; 7 Department of Epidemiology, MD Anderson Cancer Center, University of Texas, Houston, Texas, United States of America; 8 Affymetrix Inc., Santa Clara, California, United States of America; 9 Department of Systems Biology, MD Anderson Cancer Center, University of Texas, Houston, Texas, United States of America; University of Chicago, United States of America

## Abstract

A number of studies of copy number imbalances (CNIs) in breast tumors support associations between individual CNIs and patient outcomes. However, no pattern or signature of CNIs has emerged for clinical use. We determined copy number (CN) gains and losses using high-density molecular inversion probe (MIP) arrays for 971 stage I/II breast tumors and applied a boosting strategy to fit hazards models for CN and recurrence, treating chromosomal segments in a dose-specific fashion (-1 [loss], 0 [no change] and +1 [gain]). The concordance index (C-Index) was used to compare prognostic accuracy between a training (n = 728) and test (n = 243) set and across models. Twelve novel prognostic CNIs were identified: losses at 1p12, 12q13.13, 13q12.3, 22q11, and Xp21, and gains at 2p11.1, 3q13.12, 10p11.21, 10q23.1, 11p15, 14q13.2-q13.3, and 17q21.33. In addition, seven CNIs previously implicated as prognostic markers were selected: losses at 8p22 and 16p11.2 and gains at 10p13, 11q13.5, 12p13, 20q13, and Xq28. For all breast cancers combined, the final full model including 19 CNIs, clinical covariates, and tumor marker-approximated subtypes (estrogen receptor [ER], progesterone receptor, *ERBB2* amplification, and Ki67) significantly outperformed a model containing only clinical covariates and tumor subtypes (C-Index_ full model_, train[test]  =  0.72[0.71] ± 0.02 vs. C-Index_ clinical + subtype model_, train[test]  =  0.62[0.62] ± 0.02; p<10^−6^). In addition, the full model containing 19 CNIs significantly improved prognostication separately for ER–, HER2+, luminal B, and triple negative tumors over clinical variables alone. In summary, we show that a set of 19 CNIs discriminates risk of recurrence among early-stage breast tumors, independent of ER status. Further, our data suggest the presence of specific CNIs that promote and, in some cases, limit tumor spread.

## Introduction

Gene expression profiling, coupled with patient outcomes, has demonstrated the extent and clinical importance of molecular heterogeneity among breast cancers [1]–[4]. As a result, human breast cancers have been subclassifed into four reproducible subtypes: luminal A (LUM A), luminal B (LUM B), *ERBB2*-amplified (HER2+), and basal-like [3]. These expression-based subtypes predominantly divide on the clinical subtypes defined by immunohistochemical (IHC) measures of estrogen receptor (ER), where luminal-type tumors are predominantly ER-positive (ER+), and basal-like tumors are ER negative (ER−) [5]. Luminal-type tumors, LUM A and LUM B, can be further discriminated by differences in their proliferation indices assessed by IHC measures of Ki67 as low or high, respectively [6].

As a consequence of extensive gene expression profiling, first generation gene signature-based diagnostic tests (e.g., OncotypeDx® and MammaPrint®) have entered clinical diagnostics for patients with early-stage tumors that are non-amplified for *ERBB2*, the gene that codes for the human epidermal growth factor receptor 2 (*i.e.,* HER2– breast cancers) [7]. These early gene signatures largely stratify patients on known clinical factors, showing improved quantitation and reproducibility for measures of hormone receptor status and proliferation over routine IHC testing. While these molecular tests show improved reproducibility for risk classification for a subgroup of patients, the gains in prognostication over clinical models are fairly modest, with little to no discrimination for tumors that are ER-low, ER−, HER2+, or histologically advanced at diagnosis; all of which exhibit heterogeneity in terms of patient outcomes [4].

A number of studies demonstrate the coupling of chromosomal abnormalities as copy number imbalances (CNIs) with the gene expression-based tumor subtypes [8]–[13], and, in many cases, specific CNIs have been shown to directly influence gene expression [9], [14]–[16]. Importantly, there is also evidence of sharing of specific genomic alterations across the expression-based subtypes, some of which seem to be particularly important drivers for tumor aggressiveness. For example, Chin *et al*. found that high-level amplifications and chromosomal alterations at 8p11-12 and 11q13-14, which were strongly associated with poor outcomes, were present in all expression-based subtypes but at different frequencies [9]. Because tumor cells that lose genomic stability acquire a number of secondary somatic mutations and chromosomal alterations that include CNIs, we hypothesize that some of these changes, perhaps under shared selective pressures, directly influence metastatic potential independent of the expression subtypes and, if identified, may aid in further refining patient prognostication.

The association between genome-wide CNI profiles and breast cancer outcomes is limited to a handful of highly promising investigations [8]–[9], [17]. Thus far, however, only measures of the *ERBB2* gene amplicon coding for HER2 have entered the diagnostic setting and solely for selecting patients for targeted therapy with Herceptin® [18]. More recent efforts have focused on associations within tumor subtypes. For example, the RAB11 family-interacting protein 1 gene (*RAB11FIP1*), which codes for a RAB-coupling protein [RCP] and the putative driver of the 8p11-12 amplicon, has been associated with poor outcomes in LUM B patients particularly when co-amplified with the Ras-related protein Rab25 gene (*RAB25*) at 1q22 [19]–[20]. While promising, such studies are limited by small numbers of cases and inclusion of predominantly larger, more-advanced-stage breast tumors for which fresh frozen material was available. Investigation of larger sample sets of early-stage tumors with long-term follow-up is absent, largely as a result of tissues stored as formalin-fixed, paraffin-embedded (FFPE) blocks and the challenges associated with deriving copy number (CN) information from FFPE materials [21].

To overcome the inherent challenges in using FFPE tumor tissues in CN determination, we applied high-density molecular inversion probe (MIP) arrays to characterize CN status in 971 stage I/II breast cancers as FFPE. We report specific CNIs identified through a boosting strategy [22] and their independent and combined use with clinical covariates and tumor subtypes in predicting recurrence risk. Our results support the integration of specific CNIs in prognostication of early-stage breast cancers and separately for tumors that are LUM B, ER−, or HER2+. In addition, we show that modeling CNIs in a gene-dose fashion identified specific chromosomal regions whose gain or loss demonstrated opposing effects on recurrence risk.

## Results

### Molecular inversion probe (MIP)-determined CNIs and their association with tumor marker-defined subtypes

**Figure 1:**
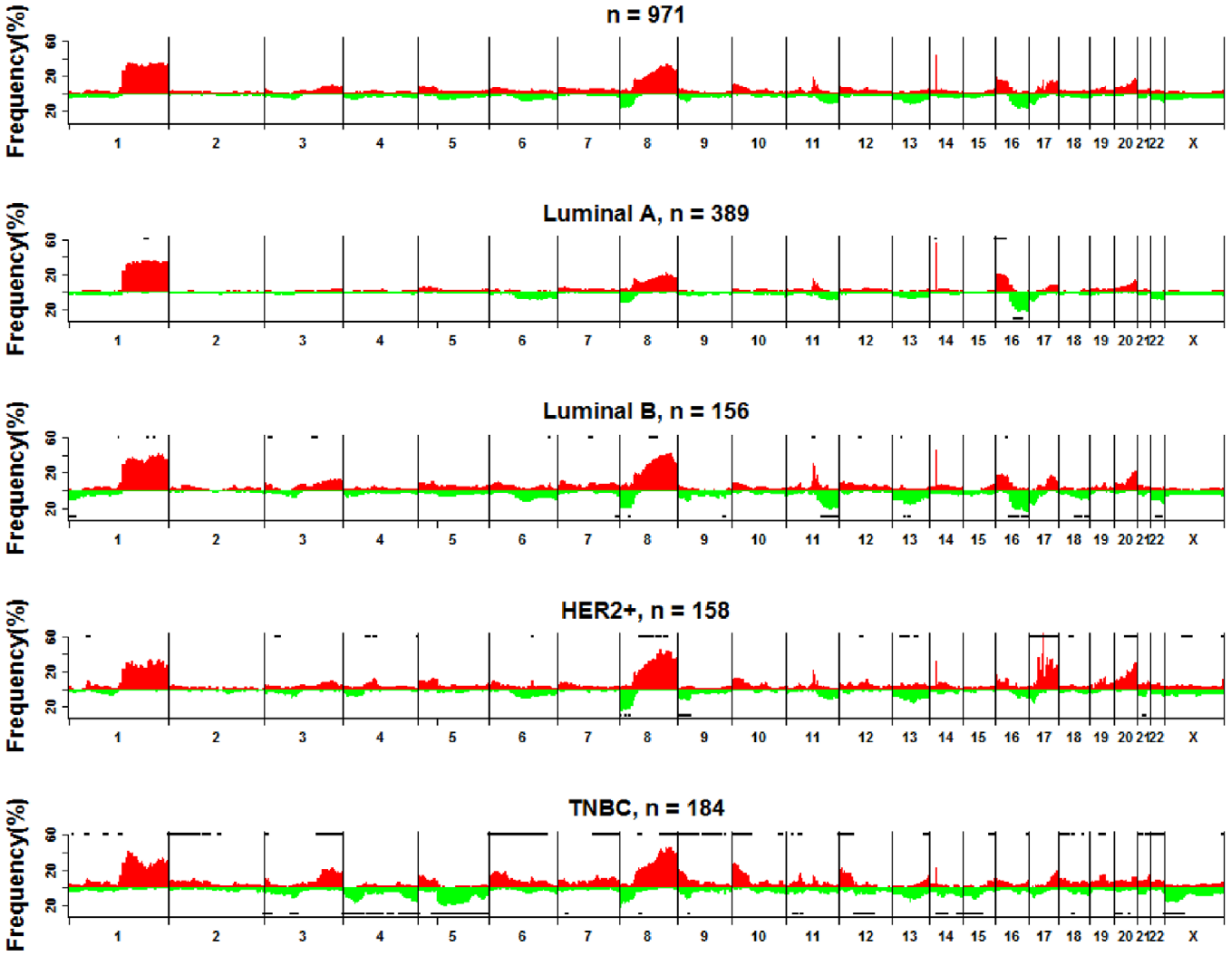
The five panels show the percentage of samples showing gain (red) or loss (green) for all 971 tumors (top) and individually for each clinical subtype. The horizontal black lines at the top (and bottom) of a panel associated with a particular clinical subtype indicate regions showing statistically significant increase in gain (and loss) frequencies (FDR<0.01) for this subtype compared with the other subtypes.

CNIs were determined using MIP-based arrays for stage I/II breast tumors from 971 patients, whose clinical characteristics are described in Table 1. Figure 1 shows the pattern of CNIs for all 971 tumors and by subtype defined as LUM A, LUM B, HER2+, and triple negative breast cancer (TNBC) that were approximated using tumor markers as described in Materials and Methods. Using MIP arrays, we found a pattern of recurrent (≥10%) gains and losses in early-stage breast tumors (all combined and by tumor subtype) that were consistent with those previously described from studies using comparative genome hybridization of fresh frozen tumors [8], [23]. These are shown in Figure 1 with detailed annotation by subtype provided in Table S1. For example, all tumor subtypes showed recurrent gains of the 1q arm as well as gains of 8p11.23-q24.3, 11q13.2-q13.3, 14q11.2, and 20q13.13-q13.33 with recurrent losses at 8p23.3-p12. The 41 recurrent CNIs that differ significantly at a false discovery rate (FDR) of 0.01 across the tumor subtypes are indicated in Table S1. As reported by others [8], gains of 16p13.3-p11.2 and losses of 16q12.1-q24.3 were more common in LUM tumors, whereas losses of 1p36.23-p36.31, 6q14.1-q27, 11q14.1-q25, and 22q11.1-q13.33 were significantly more common among LUM B tumors. Furthermore, gains at 4q13.3-q21.21 and 17q11.1-q23.2, which includes the *ERBB2* amplicon, were more common among HER2+ tumors. When separated on ER status (Figure S1), HER2+/ER+ tumors were similar to HER2+/ER– tumors for the extent and type of CNIs, with the exception of a significantly higher proportion of HER2+/ER+ tumors exhibiting gains at 17q distal to the *ERBB2* locus (45.7%) and gains at 8p12 (28.4%), compared with HER2–/ER– tumors (20.8% and 12.2%, respectively; FDR<0.05). When separated on ER status (Figure S1), HER2+/ER+ tumors were similar to HER2+/ER– tumors for the extent and type of CNIs, with the exception of a significantly higher proportion of HER2+/ER+ tumors exhibiting gains at 17q distal to the ERBB2 locus (45.7%) and gains at 8p12 (28.4%), compared with HER2–/ER– tumors (20.8% and 12.2%, respectively; FDR<0.05). In addition, using a relaxed FDR<0.1 for exploratory purposes given the small sample size, gains at 5q35.1, 8p12, 10q21.1, and 17q11.2-q25.2 and losses at 6q14.1-q22.31, 6q27, 9q21.13-q33.1, 11q14.1-q22.3, 13q12.3 and 17p13.1 were more common in HER2+/ER+ when compared to HER2+/ER–. TNBCs showed numerous recurrent CNIs including losses at 3p12.3-p12, 14q13.3-q32.31, 15q12-q14, and Xp22.21-p11.23, and gains at 1p12, 6p25.3-p12.1, 6q16.2-q23.1, 7q22.1-q35, 9p24.3-p21.3, 11p13-p12, 12p13.33-p11.2, 13q33.3-q34, 18p11.32-p11.21, and 21q22-q22.3. Consistent with prior studies for basal-like tumors [9], TNBCs exhibited extensive losses on chromosome 4 (4p16.1-q35.2) and losses on the 5q arm. Recurrent gains at 5p15.33-p13.1 and 17q23.2-q25.3, and losses at 9p21.2-p21.1, 13q14.2-q31.1, and 17p12 were present among LUM B, HER2+, and TNBC tumors, but not the LUM A group.

**Table 1:** Clinical characteristics of all stage I/II breast cancer patients with MIP derived copy number.

Characteristic	White	Black	Hispanic	Other	Total
	(N = 715)	(N = 125)	(N = 123)	(N = 8)	(N = 971)
**Age (yrs), mean (s.d.)**	55 (12.7)	54 (13.8)	52.1 (10.9)	51.8 (8.7)	54.4 (12.6)
**Year of Diagnosis**					
≥1995	304 (42.5)	45 (36)	56 (45.5)	0	405 (41.7)
1990–1994	227 (31.8)	4 2(33.6)	38 (30.9)	0	307 (31.6)
1985–1989	174 (24.3)	36 (28.8)	27 (22)	0	237 (24.4)
Missing	10 (1.4)	2 (1.6)	2 (1.6)	8 (100)	22 (2.3)
**Stage, # (%)**					
I	224 (31.4)	33 (26.4)	47 (38.2)	0	303 (31.2)
IIa	337 (47.1)	54 (43.2)	49 (39.9)	1 (12.5)	441 (45.4)
IIb	151(21.1)	37(29.6)	26(21.1)	7(87.5)	221(22.8)
Missing	3(0.4)	1(0.8)	1(0.8)	0	5(0.01)
**Tumor Subtype** 1 **, # (%)**					
LUM A	310 (43.3)	33 (26.4)	45 (36.6)	1 (12.5)	389 (40.1)
LUM B	110 (15.4)	23 (18.4)	19 (15.5)	4 (50)	156 (16.1)
Her2	107 (15)	25 (20)	24 (19.5)	2 (25)	158 (16.4)
TNBC	123 (17.2)	34 (27.2)	26 (21,1)	1 (12.5)	184 (18.8)
Missing	65 (9.1)	10 (8)	9 (7.3)	0	84 (8.6)
**Nuclear Grade** 2 **, # (%)**					
1–2	434 (60.7)	60 (48)	75 (61)	0	569 (58.6)
3	234 (32.7)	58 (46.4)	44 (35.8)	0	336 (34.6)
Missing	47 (6.6)	7 (5.6)	4 (3.2)	8 (100)	66 (6.8)
**Tumor size, # (%)**					
<2cm	419 (58.6)	58 (46.4)	89 (72.4)	0	566 (58.3)
≥2 cm	276 (38.6)	62 (49.6)	31 (25.2)	0	369 (38.0)
Missing	20 (2.8)	5 (4)	3 (2.4)	8 (100)	36 (3.7)
**Lymph node, # (%)**					
0	409 (57.2)	82 (65.6)	74 (60.2)	0	565 (58.2)
≥1	295 (41.3)	41 (32.8)	47 (38.2)	0	383 (39.4)
Missing	11 (1.5)	2 (1.6)	2 (1.6)	8 (100)	23 (2.4)
**Endocrine treatment**					
No	382 (53.4)	74 (59.2)	66 (53.7)	0 (0)	522 (53.8)
Yes	320 (44.8)	47 (37.6)	55 (44.7)	0 (0)	422 (43.5)
NA	13 (1.8)	4 (3.2)	2 (1.6)	8 (100)	27 (2.7)
**Chemotherapy**					
No	353 (49.4)	68 (54.4)	59 (48)	0 (0)	480 (49.4)
Yes	327 (45.7)	51 (40.8)	59 (48)	0 (0)	437 (45)
NA	35 (4.9)	6 (4.8)	5 (4.1)	8 (100)	54 (5.6)
**Radiation therapy**					
No	397 (55.5)	73 (58.4)	65 (52.8)	0 (0)	535 (55.1)
Yes	305 (42.7)	49 (39.2)	56 (45.5)	0 (0)	410 (42.2)
NA	13 (1.8)	3 (2.4)	2 (1.6)	8 (100)	26 (2.7)

1 Tumor subtype defined by ER, PR, Ki67 and HER2 as described in Materials and Methods.

2 Nuclear grade was determined by the Modified Black's method.

### Specific CNIs improve prognostication for any recurrence, distant metastasis, and overall survival

To assess the prognostic information of individual CNIs obtained across the whole genome, we built a Cox proportional hazards model for recurrence from the high-dimensional segment data using a training set (n = 723) and the CoxBoost algorithm [24]. Using this strategy, we identified 19 specific CNIs that combined were significantly associated with risk of recurrence. We compared the performance of the 19-CNI ‘signature’ to a ‘clinical’ model that included patient age at diagnosis, lymph node status, and tumor size and a ‘clinical + subtype’ model that included clinical covariates combined with tumor subtypes (*i.e.,* LUM A, LUM B, TNBC, and HER2+). The development of the different multivariate models, including the variable selection approach for the CNIs, is detailed in Materials and Methods.

**Figure 2:**
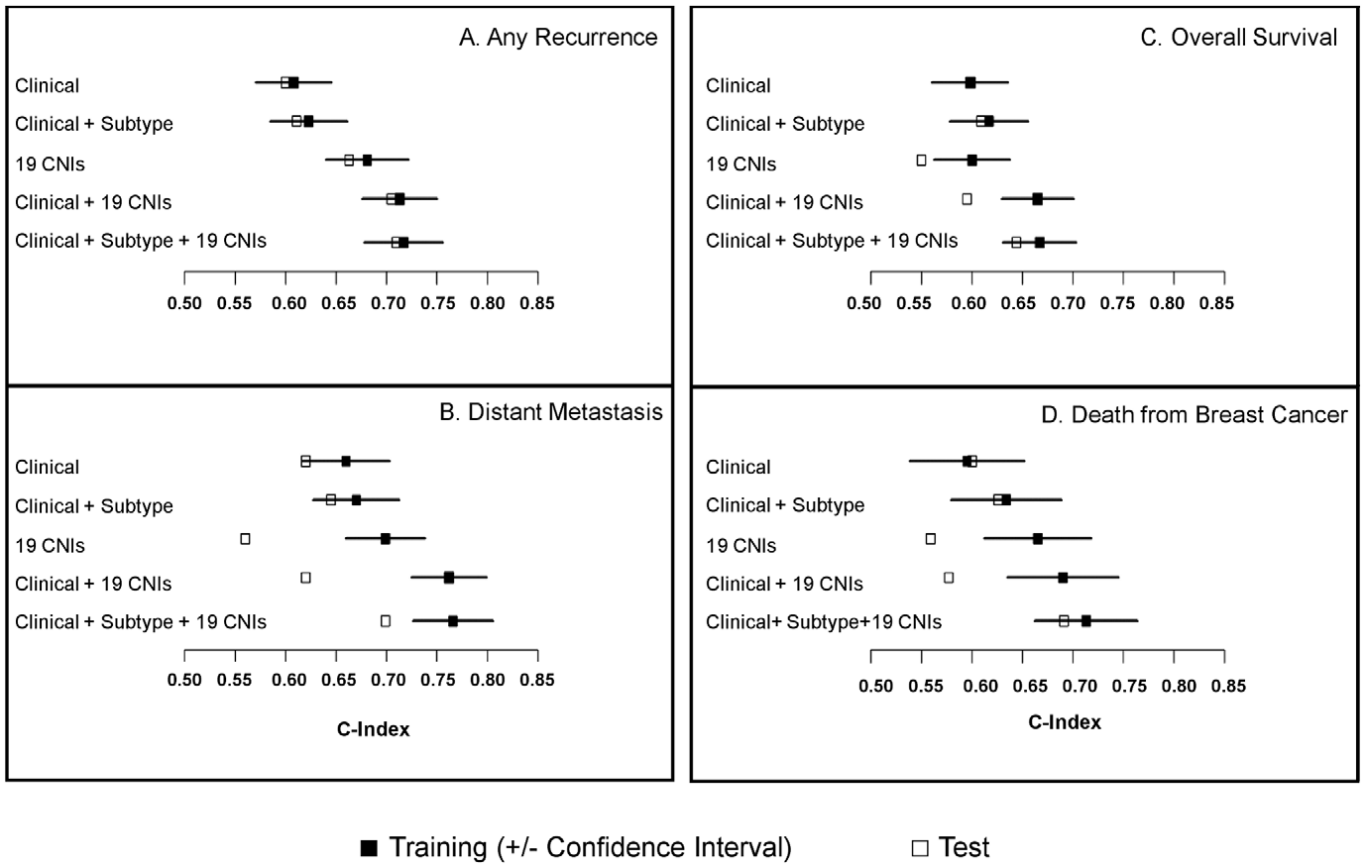
The performance of the clinical, 19-CNI, combined (clinical + tumor subtype) and full (19-CNIs, clinical, tumor subtype) prognostic models by Concordance Index: (A) recurrence, (B) distant metastasis, (C) overall survival, and (D) death from breast cancer. Concordance indices of prognostic models for the various outcomes are shown for the training and test set. The closed square indicates the training set with the 95% confidence interval for the estimate, and the open square represents performance in the test set.

When applied across all breast cancers for any recurrence (Figure 2A), the 19-CNI model is a significantly (p<0.001) stronger predictor for recurrence (Concordance Index [C-Index] ± standard error  =  0.68±0.03) than either the clinical model (C-Index  =  0.61±0.02) or the clinical + subtype model (C-Index  =  0.62±0.02). We next evaluated the performance of a ‘full’ model that included the 19 CNIs, clinical covariates, and tumor subtypes. Though not significantly different from the 19-CNI model alone (p = 0.13), the full model performed the best in both the training (C-Index  =  0.72±0.02) and test (C-Index  =  0.71) sets.

To evaluate the prognostic value of the 19 CNIs for additional patient outcomes, we next compared the four models (19-CNI, clinical, clinical subtype, and full) for time-to-distant metastasis, overall survival, and death from breast cancer (Figure 2B–D). For time-to-distant metastasis (n = 208 events), the full model (C-Index  =  0.76±0.04) performed significantly better (p = 0.01) when compared with the clinical model (C-Index  =  0.66±0.04) and clinical subtype model (C-Index  =  0.68±0.041). For overall survival, the final combined model outperformed both clinical models with and without tumor subtypes (p = 0.01) with a similar, but not significant (p = 0.18), improvement for death due to breast cancer (n = 149 deaths). The features of the full multivariate model are shown in Table 2 with more detailed information on the 19 CNIs and the genes contained within the segments provided in Table S2.

**Table 2:** Full Multivariate Cox model, based on the training set (n = 728)^1^.

Factor		N (N_recurrence)	Hazard Ratio (95% CI)	P value
**Age at Dx, yrs**				
>50		414 (115)	1	
≤50		295 (109)	1.13 (0.84–1.51)	0.42
Missing		19 (9)		
**Tumor size, cm**				
≤2		422 (116)	1	
>2		277 (103)	1.15 (0.85–1.55)	0.36
Missing		29 (14)		
**Lymph node status**				
0		422 (106)	1	
≥1		286 (117)	2.04 (1.45–2.78)	<0.001
Missing		20 (10)		
**Subtype**				
LUM A		285 (78)	1	
LUM B		110 (47)	1.50 (1.02–2.22)	0.015
HER2		125 (39)	1.03 (0.67–1.57)	0.90
TNBC		142 (48)	1.3 (0.85–1.99)	0.22
Missing		66 (21)		
**Cytoband**	**Start-Stop**	**Gain or Loss**		
1p12	nt119315210-nt119747280	loss	1.96 (1.10–3.45)	0.02
2p11.1	nt91087616-nt94286916	gain	2.18 (0.90 – 5.45)	0.09
3q13.12-q13.13	nt108059123-nt112251638	gain	4.35 (2.42 – 7.82)	<0.001
8p22	nt17229368-nt17457649	loss	1.52 (1.09 – 2.08)	0.016
10p11.21	nt36379031-nt37813659	gain	0.24 (0.11 – 0.53)	<0.001
10p13	nt16084814-nt17528387	gain	2.03 (1.22 – 3.39)	0.007
10q23.1	nt82273705-nt82913296	gain	1.52 (0.87 – 2.66)	0.14
11p15.1-p15.2	nt14183576-nt19267810	loss	0.59 (0.31 – 1.12)	0.11
11q13.5	nt75779338-nt76296812	gain	1.44 (0.94 – 2.20)	0.09
12q13.13	nt50493755-nt51600159	loss	1.96 (0.98 – 3.85)	0.06
12p13.32	nt3394093-nt3630092	gain	1.75 (1.07 – 2.84)	0.03
13q12.3	nt28554115-nt29380652	loss	0.40 (0.24 – 0.68)	<0.001
14q13.2-q13.3	nt35380230-nt36252346	gain	1.82 (1.13 – 2.93)	0.02
16p11.2	nt31526202-nt35843070	loss	1.54 (0.98 – 2.38)	0.06
17q21.33	nt47411130-nt48137311	gain	0.31 (0.18 – 0.54)	<0.001
20q13.33	nt59456751-nt59788832	gain	1.27 (0.88 – 1.83)	0.21
22q11.1-q11.21	nt15236255-nt16625906	loss	1.82 (1.10 – 3.01)	0.02
Xp21.1-p21.2	nt30907133-nt32653344	loss	2.78 (1.72 – 4.55)	<0.001
Xq28	nt151081086-nt151871524	gain	1.87 (1.19 – 2.94)	0.007

1 The clinical covariates shown were selected from a step-wise model selection procedure that minimizes the Akaike Information Criteria, except for age at diagnosis.

### Prognostic CNIs, recurrence, and frequency by tumor subtype

With the selection of the 19 CNIs, we confirm previous studies reporting higher risk of recurrence among breast tumors exhibiting losses at 8p22 and 16p11.2, and gains at 10p13, 11q13.5, 12p13, 20q13, and Xq28 [8]-[9]. In addition, we identified 12 CNIs not previously associated with breast cancer recurrence: 1p12, 2p11.1, 3q13.12, 10p11.21, 10q23.1, 11p15, 12q13.13, 13q12.3, 14q13.2-13.3, 17q21.33, 22q11, and Xp21. Figure S2 shows the time-to-recurrence for each of the individual CNIs. Consistent with the previous report for CNIs [9], the CNIs in the 19 segments were present across all IHC-defined subtypes but differed significantly in frequency by subtype (Table S3). For example, gains at 1p12, 2p11.1, and 10p13, and losses at Xp21.1, were more common (p<0.001) in TNBCs, whereas gains at 17q21.33 and 20q13.33 were more common (p<0.001) among LUM B and HER2+ tumors.

### CNI model improves prognostication within tumor subtypes

**Figure 3:**
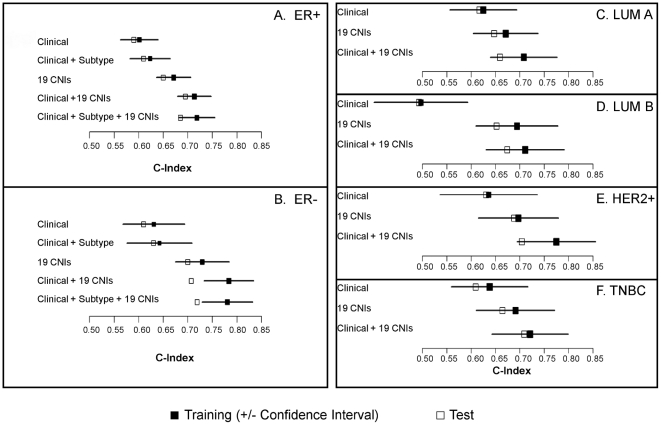
Concordance indices of prognostic models for probability of recurrence in training and test Set: (A) ER+, (B) ER-, (C) LUM A, (D) LUM B, (E) HER2+, and (F) TNBC. The closed square indicates the training set with the 95% confidence interval for the estimate, and the open square represents performance in the test set.

When separated on ER status (Figure 3A and 3B) the 19 CNI model significantly improved prognostication when compared with the clinical plus tumor subtypes: ER+ (C-Index  =  0.72 vs. 0.62, p<0.0001) and ER– (C-Index  =  0.78 vs. 0.63, p = 0.001). Further, when assessed within each tumor subtype group separately, the 19 CNIs plus clinical covariates (age of diagnosis, lymph node status, tumor size) showed improved prognostication across all subtypes (Figure 3C-F) compared with the clinical model: LUM A (C-Index  =  0.71 vs. 0.63, p = 0.047), LUM B (C-Index  =  0.71 vs. 0.50, p = 0.002), any HER2+ (C-Index  =  0.78 vs. 0.64, p = 0.014), and TNBC (C-Index  =  0.72 vs. 0.64, p = 0.046).

### Risk index based on CNIs and recurrence probability

**Figure 4:**
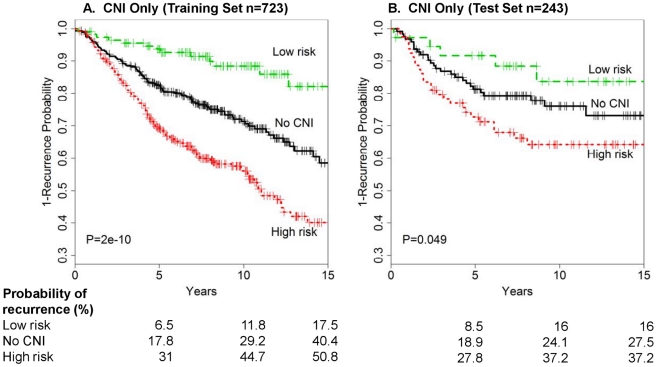
Time-to-recurrence for all breast cancers by 19 CNI-only risk groups. Risk groups are defined as low-risk CNIs, no CNIs at the 19 markers (no-CNIs), and high-risk CNIs: (A) training set and (B) test set.

To classify individuals based on the 19 CNIs alone and to gain some insight on how information on the 19 CNIs relates to clinical characteristics, we next created risk categories of ‘low’, ‘no CNI’ (no CNIs in any of the 19 segments), and ‘high’, as described in Materials and Methods. Figure 4 shows the recurrence probability for all breast cancers classified as low (15.8%), no-CNI (46.2%), or high (38%) risk for both the training (Figure 4A) and test (Figure 4B) sets. In the training set, the probability of recurrence was greatest for patients presenting with the high-risk CNI signature. For example, among the high-risk group, 31% recurred by 5 years compared with 6.5% of the low-risk patients. Patients showing no imbalances in the 19 CNIs experienced intermediate risk, with 17.8% recurring in the same time periods. Compared with the patients in the no-CNI group, those in the high-risk CNI group had a significant increase in risk of recurrence [hazard ratio (HR)  =  1.8; 95% confidence interval (CI), 1.37-2.36], whereas those classified into the low-risk CNI group had significantly lower risk HR  =  0.39 (0.23–0.69).

Table 3 shows the association between the clinical characteristics and the prognosis signature for the CNI-defined risk groups. There were no differences among the three groups by race/ethnicity, lymph node status, hormone therapy, or radiation therapy. Tumors in the low- and high-risk groups were significantly more likely to be larger, be nuclear grade III, and have ≥20% positive staining for the proliferation marker Ki67 than the no-CNI, intermediate-risk group. The two CNI-defined groups showed similar within-group occurrence of *ERBB2* amplification (∼20%). While similar to the low-risk group on a number of clinical features, the high-risk group was more likely to be ER–, receive chemotherapy, and be younger at diagnosis than the low- or intermediate-risk groups.

**Table 3:** Association Between Clinicopathological Characteristics and Marker-based Risk Signatures.

Characteristic^1^	Low Risk (n = 153)	No CNI (n = 449)	High Risk (n = 369)	P value
**Age at diagnosis (years)**				
<40	14 (9.2)	50 (11.1)	50 (13.6)	
40–50	42 (27.5)	131 (29.2)	115 (31.2)	
50–60	41 (26.8)	110 (24.5)	111 (30.1)	
>60	56 (36.6)	155 (34.5)	91 (24.7)	0.04
**Race/Ethnicity**				
White	110 (71.9)	334 (74.4)	271 (73.4)	
African American	21 (13.7)	51 (11.4)	53 (14.4)	
Hispanic	21 (13.7)	59 (13.1)	43 (11.7)	0.70
**Tumor Subtype** 1 **, no. (%)**				
LUM A	51 (33.3)	225 (50.1)	113 (30.6)	
LUM B	32 (20.9)	53 (11.8)	71 (19.2)	
HER2+	32 (20.9)	53 (11.8)	73 (19.8)	
TNBC	24 (15.7)	71 (15.8)	89 (24.1)	<0.001
**Nuclear Grade** 3 **, no. (%)**				
I/II	81(53.0)	296 (65.9)	192 (52.0)	
III	65 (42.5)	110 (24.5)	161 (43.6)	<0.001
**Tumor size, no. (%)**				
<1	14 (9.2)	84 (18.7)	31 (8.4)	
1–2	71 (46.4)	201 (44.8)	165 (44.7)	
>2	63 (41.2)	141 (31.4)	165 (44.7)	<0.001
**Lymph node, no (%)**				
0	87 (56.9)	268 (59.7)	210 (56.9)	
≥1	62 (40.5)	167 (37.2)	154 (41.7)	0.50
**ER status**				
Negative	41 (26.8)	114 (25.4)	138 (37.4)	
Positive	110 (71.9)	331 (73.7)	225 (61)	<0.001
**Ki67 status (%)**				
<20	63 (41.2)	277 (61.7)	155 (42)	
≥20	69 (45.1)	106 (23.6)	176 (47.7)	<0.001
**HER2 status**				
Negative	121 (79.1)	307 (88.7)	296 (80.2)	
Positive	32 (20.9)	39 (11.3)	73 (19.8)	0.002
**Endocrine Therapy**				
No	76 (49.7)	236 (52.6)	210 (56.9)	
Yes	72 (47.1)	198 (44.1)	152 (41.2)	0.34
**Chemotherapy**				
No	84 (54.9)	234 (52.1)	162 (43.9)	
Yes	62 (40.5)	184 (41)	191 (51.8)	0.008
**Radiation therapy**				
No	87 (56.9)	226 (50.3)	222 (60.2)	
Yes	62 (40.5)	206 (45.9)	142 (38.5)	0.24
**1q**				
No Gain	86 (56.2)	340 (75.7)	202 (54.7)	
Gain	67 (43.8)	109 (24.3)	167 (45.3)	<0.0001
**16p**				
No Gain	116 (75.8)	409 (91.1)	274 (74.3)	
Gain	37 (24.2)	40 (8.9)	95 (25.7)	<0.0001
**16q**				
No Loss	117 (76.5)	406 (90.4)	290 (78.6)	
Loss	36 (23.5)	43 (9.6)	79 (21.4)	<0.0001

1 Data for samples missing a specific characteristic are not shown, refer to Table 1.

2 Tumor subtype defined by ER, PR, Ki67 and HER2 as described in Materials and Methods.

3 Nuclear grade was determined by the Modified Black's method.

**Figure 5:**
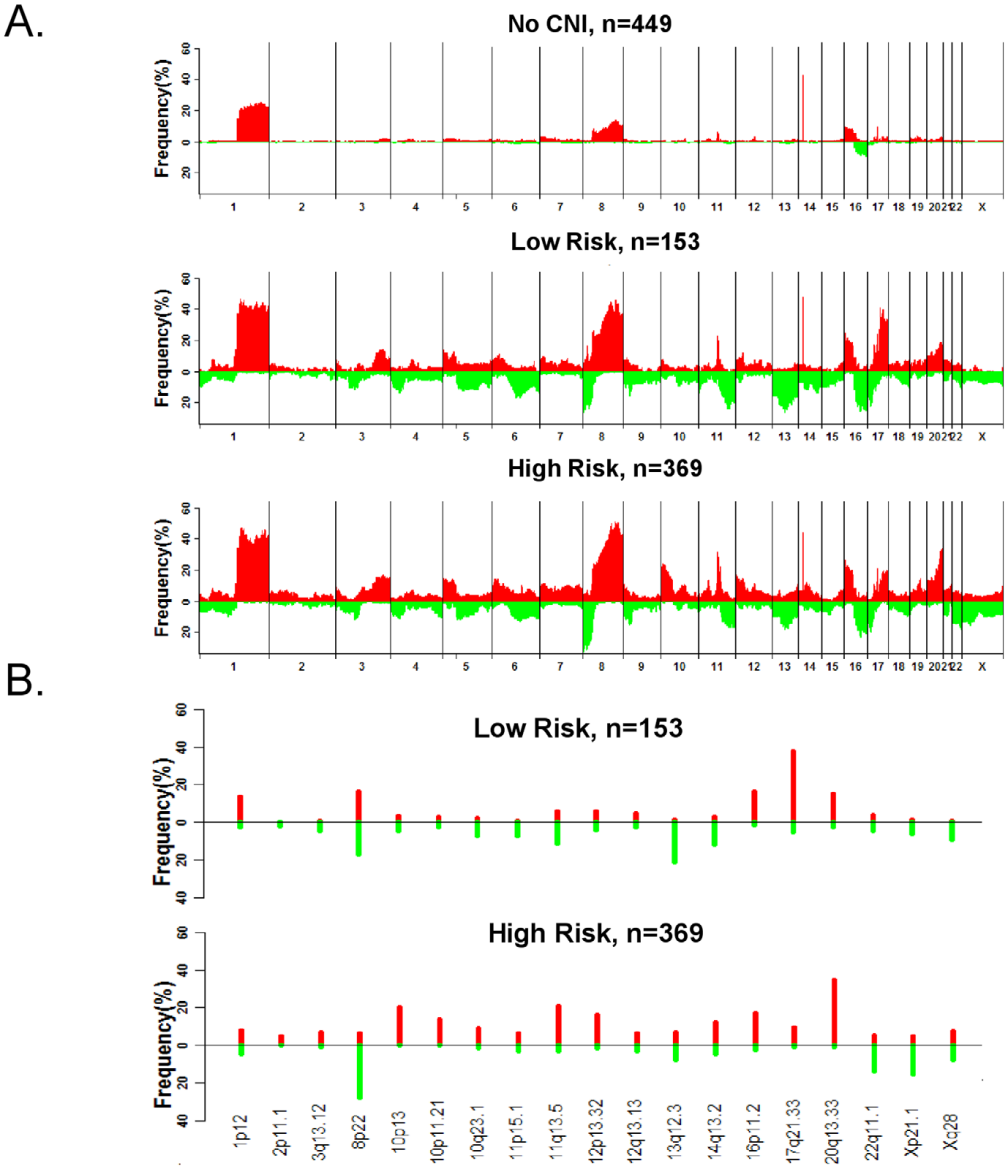
Pattern of copy number imbalances and their frequency across risk subtypes. (A) Frequency and type of CNIs (red,gain; loss,green) across the entire genome for the three marker-based risk groups (low-risk CNIs, no-CNIs, and high-risk CNIs). (B) CN gain/loss frequencies for the 19 CNIs and for the low- and high-risk CNI defined groups.

The frequency and nature of gains and losses across the three risk groups are shown in Figure 5A. Both the low- and high-risk group display an overall pattern of greater gains and losses than the no-CNI group. Next, we assessed the association of the groups with the previously described ‘simplex’ tumors (*i.e.,* tumors enrichment for gains at 1q and 16q and over-represented in LUM A) [22]. Consistent with a general defect in genomic stability, both the low- and high-risk CNI groups (Table 3) showed significantly higher representation of gains at 1q and 16q as well as loss at 16p (p<0.001), suggesting that the discrimination between the high-risk and no-CNI group is not simply driven by enrichment of ‘simplex’ tumors in the no-CNI group. When compared across the 19 CNIs that define the three groups, the high-risk group displayed a greater overall pattern of amplification in the 19 CNIs compared with the low-risk group (Figure 5B), which suggest the high-risk group may be enhanced for oncogenes as putative driver events.

### CNI-based risk index identifies a low-risk group among ER− patients

**Figure 6:**
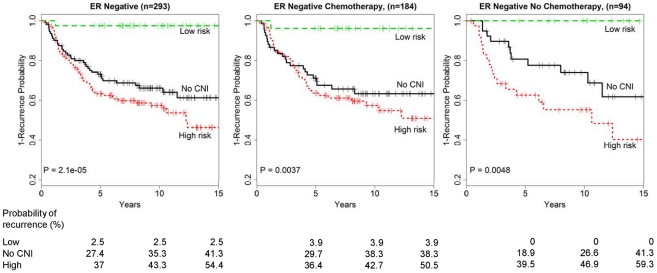
Kaplan-Meier analysis of the recurrence probability by *CNI-only* risk categories for ER− cases by treatment. **(A) all; (B) received chemotherapy; and (C) no chemotherapy.**

Consistent with a more aggressive biology, patients with ER− tumors had a significantly higher risk of recurrence [HR = 1.3 (1.01-1.64), p = 0.04] compared with ER+ cases. In our comparison of models, the 19-CNI model showed a significant improvement in prognostication for ER− tumors over clinical covariates (C-Index  =  0.78 vs. 0.63, p = 0.001) (Figure 3B). To explore the importance of the 19-CNI model for ER− tumors, recognizing treatment heterogeneity, we assessed the performance of the 19 CNI-based risk score among ER− cases by chemotherapy (Figure 6). There is a strong relationship between risk score and time-to-recurrence in ER− patients. For the CNI-based models, ∼14% of ER− cases experienced a very low hazard of recurrence [HR = 0.06 (0.01-0.42), p = 0.005] relative to the group with none of the 19 CNIs, independent of treatment with chemotherapy. While limited to a small sample size, these data suggest that women with the low-risk signature may not benefit from the addition of chemotherapy. As for the effect of chemotherapy for ER− and ‘high-risk’ individuals, a comparison of the Kaplan Meier curves by chemotherapy (Figure 6B and 6C) showed no significant differences for the ER−, high-risk group stratified by whether or not they received chemotherapy (log-rank test, p = 0.248). Further efforts with larger sample sizes are needed to determine whether or not the CNI-based classifier is informative for predicting treatment outcomes within the ER− subgroup.

### Prognostic accuracy of the CNI-based models and recurrence probability at 5 and 10 years of follow-up

Using data for all breast cancers, time-dependent receiver operator characteristic (ROC) curves were derived along with the area under the curve (AUC) for 5- and 10-year recurrence probability (Figure S3A and S3B) for four models: clinical model (clinical covariates only), clinical + subtype model (clinical covariates + tumor subtypes), marker model (19-CNIs), and full model (19 CNIs, clinical covariates, and tumor subtypes). The average AUC for the final full model was 0.71 (0.68) at 5 (or 10) years, compared with 0.65 (0.61) for the next-best clinical + subtype model and 0.65 (0.64) for the marker model. For ER– tumors (Figure S3C and S3D), the average AUC for the full model was 0.74 (0.73) at 5 (or 10) years, compared with 0.63 (0.64) for the clinical model and 0.72 (0.70) for the marker model. These results illustrate the potential contribution of CNIs for improved prognostic accuracy, particularly among ER– cases.

### Seven CNIs show posterior probabilities consistent with a strong positive effect on recurrence endpoints

Next, we used CoxBoost to construct multivariate Cox models with 19 CNIs using 100 boosting steps. Because any model selection algorithm and the final model for inference ignores the uncertainty in model selection [25], we applied Bayesian Model Averaging (BMA) as an approach to address the issue of model selection uncertainty as described in Materials and Methods. Table S4 shows the posterior probabilities for the 19-CNI model. Of the CNIs selected with 100 boosting steps, 7 of 19 (3q13.12, 10p13, 11q13.5, 14q13.2, 22q11.1, Xp21.1, and Xq28) met the criteria for strong positive effect in the model for recurrence, with 5 of these 7 (10p13, 11q13.5, 22q11.1, Xp21.1, and 3q13.12,q13.13) exerting *very* strong positive effects in the models.

### Batch effects and CNI prognostic model performance

Since measures of CN were obtained in 5 individual runs over the course of the study and during the development of the novel MIP measures of CN, we evaluated the effect of batch on the performance of our models. Across batches model performance varied, with C-Indices ranging from 0.72 to 0.93 (data not shown). Thus, a batch effect likely influenced model performance, suggesting that additional improvements in measurement precision may enhance predictability.

## Discussion

Using data obtained from early-stage breast cancer patients diagnosed from 1985-1999 with an average follow-up of 8.9 years, we identified 19 CNIs as a signature that showed reproducible improvements for prognostic accuracy of breast cancer recurrence over known prognostic clinical variables, tumor marker-approximated subtypes, or their combination. The 19-CNI model showed the greatest gains in discriminating risk of recurrence among the ER− tumors and, separately, for the LUM B and HER2+ subgroups. The CNI model performed well both within and among the tumor subtypes, supporting prior observations that a sizable number of breast tumors share features of more than one of the clinical- or expression-based subtypes [26].

Our findings extend prior evidence for losses at 8p22 and Xp21 and worse outcomes for early-stage disease, independent of tumor subtype [27]–[31]. Among chromosomal gains associated with recurrence in our cohort, several are in or near regions previously implicated in poor outcome (*e.g.*, 11q13.5 and 20q13) [13], [32], or more common among aggressive basal-like or medullary-type cancers (*e.g.*, 10p13, 12p13, and Xq28) [12], [33]–[35]. In our cohort, gains at 20q13 were present in all subtypes as a recurrent event but more common in LUM B and HER+ tumors. Unlike previous studies [13], we did not identify gains at 8q24 as an independent prognostic factor for poor outcomes among breast cancer patients. However, as reported previously by Chin *et al.*, gains at 8q24 were highly correlated with gains at 20q13 in our data even after correcting for multiple comparisons (p<0.001; data not shown). Since our procedure for marker selection elects one of any highly correlated loci, it is possible that gains at 8q24 and 20q13 are interchangeable. We did not explore the effects of interaction among coamplified CNIs in this analysis.

To our knowledge, our study is the first to report CNIs at 1p12, 2p11.1, 3q13.12, 10p11.21, 10q23.1, 11p15, 12q13.13, 14q13.2-13.3, 17q21.33, and 22q11as prognostic markers for breast cancer. Of these, loss at 22q11.1 and gains at 3q13.12 and 14q13.2-13.3 are among the 7 CNIs identified in the BMA analysis ranked as strongly positively associated with recurrence. No candidate oncogenes have been reported previously for 3q13.12 nor has this region been associated with patient outcomes. In our study, the identified region in 3q13.12 contains the stem cell genes *DDPA2* and *DDPA4* [36], which, we speculate, may contribute to more-aggressive behavior if amplified. In our patient population, the segment at 14q13 shows both a gain and loss (see Figure S2). Patients with tumors showing a gain in this locus have higher risk of recurrence than patients with no CNI at this locus. In contrast, loss of this locus is associated with improved prognosis. Gain at 14q13 has been studied in relation to lung cancer [37], and co-amplification and overexpression of the transcription factors *TTF-1*, *NKX2-8,* and *PAX9* (all located at 14q13) have been associated with cisplatin resistance in lung cancer cell lines [38]. In our sample, ∼5% of cases show amplification across an overlapping, narrow 36.2-kb region of 14q13 associated with risk of recurrence. This amplicon was present at similar frequency across the four tumor subtypes (see Figure 1 and Table S3) and is of interest as a potential modifier of treatment response.

We have less confidence in the prognostic relevance of the CNIs at 1p12, 2p11.1, 10p11.21, 10q23.1, 11p15, 12q13.13, and 17q21.33 based on results of the BMA analysis and the lack of strong prior evidence. Of these, the gain at 17q21.33 is of high interest as it is significantly more common in HER2+ (24.1%) than HER2– (6.8%) tumors (p<0.0001), consistent with published studies [39]. Hu *et al.*, [13] proposed *MYST2*, which codes for a histone acetyltransferase (HBO1) with a putative role in progesterone receptor signaling [40], as a candidate oncogene for the amplicon at 17q21 present in HER2+ tumors. To our knowledge, this is the first report showing a reduced risk of recurrence for breast tumors bearing this amplification. Additional confirmation of this amplicon, as well as the other novel CNIs identified in our selection process as prognostic markers, is warranted.

When classified based on a risk index comprised of the 19 CNIs, patients in both the high- and low-risk groups show extensive genome-level chromosomal alterations and a significantly higher proportion of high-grade tumors and greater lymph node involvement than the no-CNI group. Compared with the no-CNI group, there were significantly higher frequencies of gains at 1q and 16p and loss at 16q in the low- and high-risk groups. This is consistent with an underlying defect in maintenance of normal CN in the two groups and argues against overrepresentation of the previously described ‘simplex’ tumors [29] as an explanation for the risk differences observed across the three groups.

The characteristic of the two CNI-defined groups (low- and high-risk) contrasts with tumors lacking CNI at the 19 segments. This group displays intermediate risk of recurrence in spite of proportionally lower lymph node involvement and lower-grade disease. These results suggest that information on specific CNIs may improve prognostication over clinical covariates and tumor marker-defined subtypes, particularly among tumors exhibiting chromosomal instability that manifest as CNIs. Given the nature of the intermediate, no-CNI risk group, these results further suggest alternative alterations, not CNI and perhaps not genomic instability, as determinants of disease progression in the no-CNI group of tumors.

The selection of CNIs in our model arose from treating the segments as a dose effect (−1, 0, and +1) in the variable selection strategy and reflects, in some instances, imbalances in the same genomic loci, which appear to confer opposing effects on tumor behavior. Though individual CNIs are limited to a few events, the data shown in Figure S2 and Figure 5 suggest that some segments (*e.g.,* 2p11.1, 3q13.12, 10p13, 11q13.5, and 13q12.3) contain critical progression genes that, when lost, limit tumor metastasis.

While this study is among the largest of early-stage breast cancers, a limitation is the potential effect of misclassification using older samples and use of tumor markers to approximate the transcriptome-based tumor subtypes. It is notable that some, but not all, of the 19 CNIs have been strongly associated previously with the expression-based subtypes and are similarly associated with our tumor marker-derived subgroups (Table S3). This suggests that part of the improved prognostication in our study may result from more-direct measures of the fixed events that underlie the expression-based subtypes. Interestingly, the performance of our IHC-approximated tumor-subtype prognostic model was similar (C-Index  =  0.62) to that for transcriptome-derived intrinsic subtypes reported by Parker *et al*. [6]. Additional direct comparison efforts are needed, however, to derive and refine the best and most reproducible set of discriminatory molecular markers for clinical use in the prognostication of recurrence. Such combined approaches may be of particular importance for further risk stratification of the no-CNI group, intermediate risk group.

In summary, we have identified a set of CNIs, using archival FFPE samples and novel MIP array technology, that significantly improves risk prediction for any and distant metastasis in early-stage breast cancer, independent of IHC-defined tumor subtypes. Further, our results support the presence of gain and loss imbalances within the same genomic loci that confer opposing effects on tumor behavior, findings that may indicate important biological drivers of metastasis. The results from our model building are highly promising and support CNI measures in prognostication, particularly for refining risk classification among clinical subsets (*i.e.,* ER–, LUM B, HER2+, and TNBC) where there remains a clinical need for within-group improvement in prognostication. Further evaluation of these markers in independent replication sets, considering gene expression-derived intrinsic subtypes and treatment, is warranted.

## Materials and Methods

### Ethics Statement

This study included banked samples dating from 1985–1999 and was approved by the Institutional Review Board of the University of Texas M.D. Anderson Cancer Center (MDACC) with waiver of consent for passive follow-up of deceased patients. For those who were alive during the study period, patients were contacted and consented for study participation.

### Patient population and breast tumor specimens

Breast tumors (n = 1,003) for which we had complete clinical and follow-up data and adequate tumor DNA from FFPE tissue blocks were identified from the Early Stage Breast Cancer Repository (ESBCR) at MDACC. The cohort is a retrospective study of 2,409 women diagnosed with pathologic stage I or II breast cancer and surgically treated at MDACC between 1985 and 2000. Criteria for eligibility and cohort details have been reported previously [41]. Clinical information, including patient's age, race/ethnicity, stage, tumor size, lymph node status, nuclear grade, ER and PR status, and primary treatment, including surgery, radiation therapy, chemotherapy, and endocrine therapy, was abstracted from medical charts.

### Definition of tumor subtypes

The four mutually exclusive tumor subtypes of LUM A, LUM B, HER2, and TNBC were approximated from clinically validated IHC analyses of ER, PR, HER2, and Ki67. ER and PR status were obtained from medical records (96.6% and 95.8%, respectively) and tissue microarray studies (2.2% and 3.2%, respectively). The agreement in ER and PR status between the two sources was 84.8% and 76.4%, respectively. Data for ER and PR could not be obtained for 12 and 10 subjects, respectively. ER and PR positivity was defined as ≥1% staining. ER+/HER2– tumors were subclassified using Ki67 and a clinical threshold of ≥20% positivity into LUM A (ER+/Ki67 <20%) and LUM B (ER+/Ki67 ≥20%) [42]. HER2+ status was defined for all tumors by MIP array-based *ERBB2* CN using a threshold of 2.8 for gain. This threshold was chosen based on best fit in an ROC curve yielding an AUC of 0.94 for IHC-based HER2 measurement using clinical scoring (0 or 1, not amplified; 2+ and equivocal; 3+, amplified), see Figure S4. Sensitivity analyses using thresholds of 2.3, 2.5, and 2.8 changed the frequency of HER2+ tumors in the sample as follows: 26.9%, 21.0%, and 16.3%.

### DNA extraction

Tumor DNA was extracted from FFPE tissues and processed for CN analyses as described previously [21]. Briefly, 5–10 (5- µm) macrodissected tumor sections containing >80% tumor cells per protocol were pooled and treated three times with proteinase K in ATL Tissue Lysis Buffer™ (Qiagen, Valencia, CA). Following lysis, samples were applied to uncoated Argylla Particles™ (Argylla Technologies, Tucson, AZ) and processed according to manufacturer recommendations (http://www.argylla.com).

### Molecular inversion probe-based arrays for copy number measurement

Tumor DNA was isolated from patient tissue blocks stored as FFPE. For 129 cases, DNA from non-tumor-bearing lymph nodes, stored as FFPE, was isolated as an internal germline reference for the population. Tumor and normal DNA at 10 ng/µL was shipped to the Affymetrix™ MIP laboratory for CN measurement. The laboratory was blinded to all sample and subject information including identity of duplicates. The MIP assay has been described in detail [24], [43]–[44] including platform validation using representative, but independent, samples from the ESBCR [21]. Data from the MIP high-density arrays are deposited at the National Center for Biotechnology Information (NCBIs).

Data quality was assessed using the sample two-point relative standard error (2p-RSE), as previously described [44]. The majority (96.8%) of FFPE tumor samples applied to the MIP arrays passed the 2p-RSE threshold**.** To assess platform performance, we routinely conducted an assay quality panel check. The panel consists of 12 samples: 9 HapMap samples (including two trios), chr3X, 4X, and the UCAA812 cell line. HapMap samples were used to calculate trio concordance and genotype accuracy; male chrX defined CN = 1, 3X and 4X were used for low-CN confirmation, and UACC812 was used for high amplifications (e.g., *ERBB2* has CN = 15).

### Determination of copy number change

Data collected from 129 matched normal lymph node samples were used for normalizing the CN data; therefore, common germline CNIs have been normalized by comparing the tumors to this normal set. For each sample, we generated full-genome MIP quantifications (330K MIPs). In order to reduce the data dimension, we computed the running median within groups of 25 consecutive MIPs, yielding 13,175 data points per sample. The Circular Binary Segmentation algorithm [45]–[46] was used to convert the data to a list of segments for each sample. CN differences were analyzed with the R package DNAcopy [47], using thresholds of 2.5 for one copy gained and 1.5 for one copy lost. The parameter alpha (significance level for acceptance of change-points) used in the segmentation algorithm was set to 0.01. We recombined consecutive segments if their gain/loss calls agreed for at least 99.5% of the samples. This procedure yielded 1,593 segments, representing the entire genome. Comparisons of CN patterns across different demographic, clinical, and tumor subtype groups were performed by Fisher's exact test, chi-square test, or Wilcoxon rank-sum test, as appropriate, with random permutations of the samples to incorporate an FDR adjustment for multiple comparisons.

### Development of prediction models with copy number data

We randomly split the entire sample into two groups: 75% (n = 728) for training and 25% (n = 243) for testing. The primary endpoint of the study was time-to-any breast cancer recurrence, defined as the occurrence of local lymph node or breast recurrence; metastasis to contralateral breast, chest wall or other sites; or self-report of new breast cancer that could not be verified as a recurrence versus a second primary (n = 42). Patients not known to have a recurrence at the date of last contact were censored. Univariate Cox proportional hazards regression models were used to evaluate the associations between tumor characteristics (grade, lymph node involvement, size, and stage) and treatment (endocrine therapy, chemotherapy, radiation, and surgery) variables and time-to-recurrence.

To integrate information on CN, we applied the CoxBoost algorithm for fitting a Cox proportional hazards model with high-dimensional covariates to select CNIs relevant to recurrence [24]. It is important to note that we arbitrarily chose 100 iterations, which yielded 19 CNI markers that were used throughout model building.

Next, we used a backwards elimination procedure to fit a multivariate Cox proportional hazards model with clinical covariates, considering those that were associated with time-to-recurrence in univariate analysis (lymph node status, tumor size, and patient age). Finally, we combined the selected CNIs and clinical covariates from the above two steps with tumor subtype (LUM A, LUM B, HER2+, and TNBC) and applied backwards elimination with Cox proportional hazards modeling to derive the final multivariate model. Internal validation of this final model was performed to confirm that results were not spurious and to assess the performance of the resulting models with respect to potential overfitting. Specifically, for the training data set, we evaluated prediction performance using bootstrap .632+ estimates of prediction error curves. To assess model performance, the C-Index [48] was used to compare the strengths of the various models by fitting the same multivariate models to the test set. The C-Index is a measure of the probability of agreement between what the model predicts and the actual observed risk of breast cancer recurrence. We also used the C-Index estimates to compare differences between the individual models using a two-sample *t-*test.

### Creation of risk group classifiers

We used the coefficients of the Cox model based on the training data (n = 723) including the 19 markers as -1, 0, and +1 to define three groups: intermediate risk (tumors that show no event for the 19 markers, risk index  =  0), high risk (tumors with risk index >0), and low risk (tumors with risk index < 0).

### Time-dependent ROC curves for recurrence

We summarized the discrimination potential of our models (clinical-only, markers-only, and clinical + markers models) by calculating ROC curves for cumulative recurrence incidence at 5 and 10 years (see [49]). An ROC curve is the plot of the sensitivity versus 1-specificity of the dichotomized test *X>c* for all possible values of *c*, where *X* is a risk indicator. A time-dependent ROC curve can be produced by estimating time-dependent sensitivity and specificity:
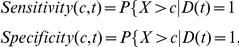
Where *D(t)* is 1 if an event (recurrence) happened up to time *t*, and 0 otherwise. For our three models, we used the log-hazard values estimated by each Cox model as a risk indicator for the ROC curve computation. We used the R package survivalROC [50].

### Bayesian Model Averaging (BMA) to address model selection uncertainty

BMA was used to examine a subset of the 2^n^ possible models (when n, the number of covariates, is large) to determine posterior probabilities of each model (see equations [25], [51]). This summation over models allows the computation of the posterior probability that the regression coefficient for a covariate is non-zero (‘posterior effect probability’), the sum of posterior probabilities of the models which contain this variable. BMA was implemented in the R package bma [52] and allows BMA for Cox models of survival [51]. Rules of thumb for the interpretation of the posterior effect probabilities are as follows: <50%, evidence against the effect; 59–75%, weak evidence for the effect; 75–95%, positive evidence for the effect; 95–99%, strong evidence for the effect, and >99%, very strong evidence for the effect.

## Supporting Information

Figure S1 **Copy number gains and losses in HER2+ tumors by ER status. (A) ER-/HER2+ and (B) ER+/HER2+.** The horizontal black lines at the top (and bottom) of a panel indicate regions showing statistically significant increase in gain (and loss) frequencies (FDR<0.01) for this subtype compared with the other subtype.
(TIFF)

Figure S2 **Time-to-recurrence using data for all breast cancers by the 19 individual copy number imbalances identified in the variable selection process.** The black line indicates no change in copy number, while green is loss and red is gain.
(TIFF)

Figure S3 **Time-dependent receiver operator characteristic (ROC) curves with the area under the curve (AUC) for the full models (19 CNIs, clinical, and tumor subtypes) compared to the clinical-only, clinical + tumor subtype, and 19-CNI ('marker only') models for 5-year (Panels A & C) and 10-year (Panels B & D) recurrence probability for all breast cancers (Panels A & B) and ER− cases only (Panels C & D).**
(TIFF)

Figure S4 **Determination of HER2 Status.** (A) log_2_(copy number) by HER2 immunohistochemistry score from 848 breast tumors in tissue microarray studies. (B) Receiver Operator Curve (ROC) for the HER2 classifier based on copy number using a threshold of 2.8 as definition for gain.
(TIFF)

Table S1 **Recurrent Copy Number Gains and Losses (≥10%) by Tumor Subtype.**
(DOCX)

Table S2 **Expanded start and stop boundaries, segment size, and genes associated with 19 copy number imbalances selected for recurrence.**
(DOCX)

Table S3 **Frequency of 19 Copy Number Imbalances by Subtype.**
(DOCX)

Table S4 **Posterior Probabilities for the individual 19 CNIS for the Full and 19 CNI Only Models.**
(DOCX)
