# Supplementary figures and images for: Selective Genomic Copy Number Imbalances and Probability of Recurrence in Early-Stage Breast Cancer
Source: PLoS One. 2011 Aug 12;6(8):e23543. doi: 10.1371/journal.pone.0023543 (PMC3155554; doi:10.1371/journal.pone.0023543)

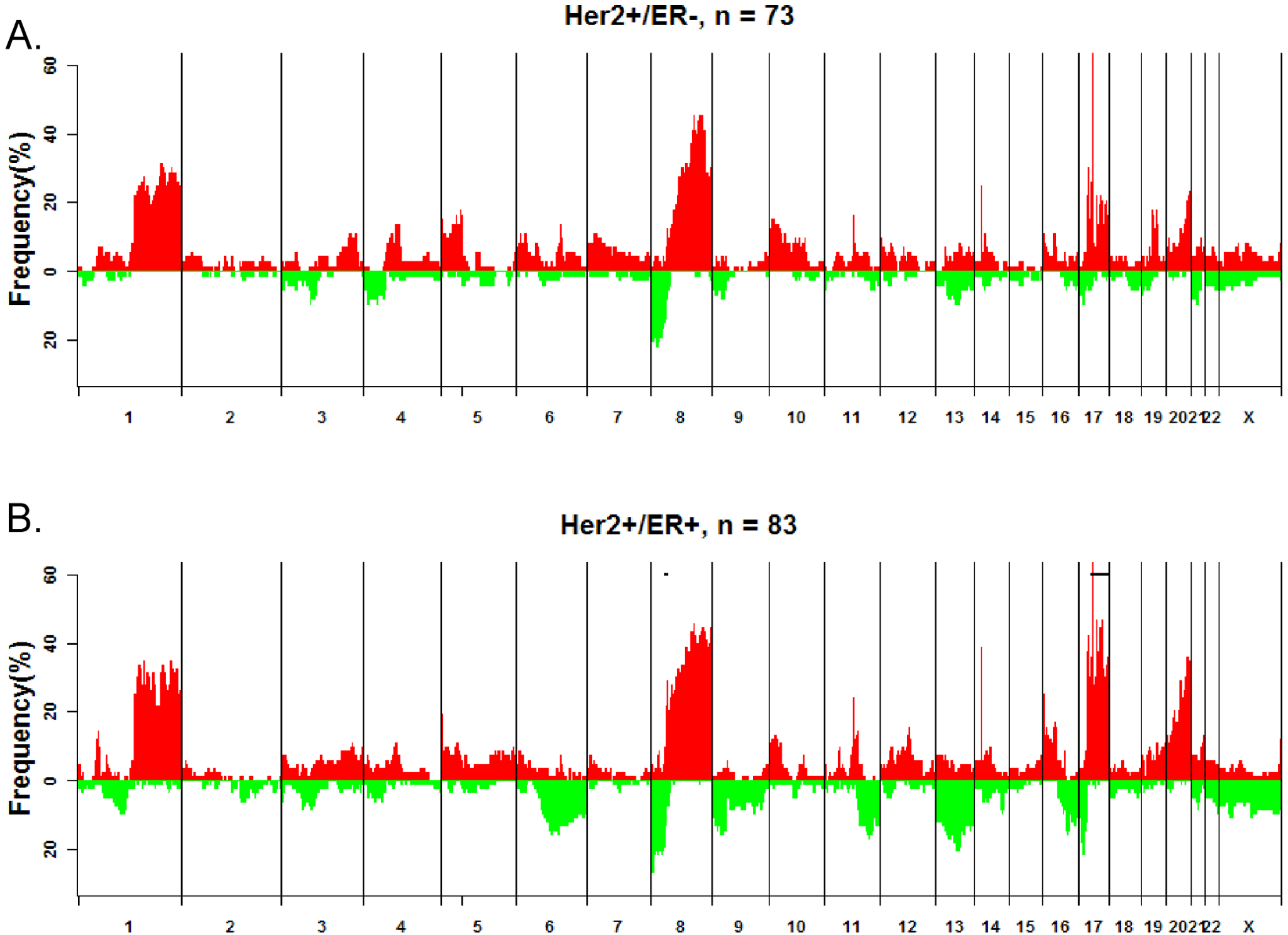

Supplement: Figure S1 — Copy number gains and losses in HER2+ tumors by ER status. (A) ER-/HER2+ and (B) ER+/HER2+. The horizontal black lines at the top (and bottom) of a panel indicate regions showing statistically significant increase in gain (and loss) frequencies (FDR<0.01) for this subtype compared with the other subtype. (TIFF) [file pone.0023543.s001.tiff]

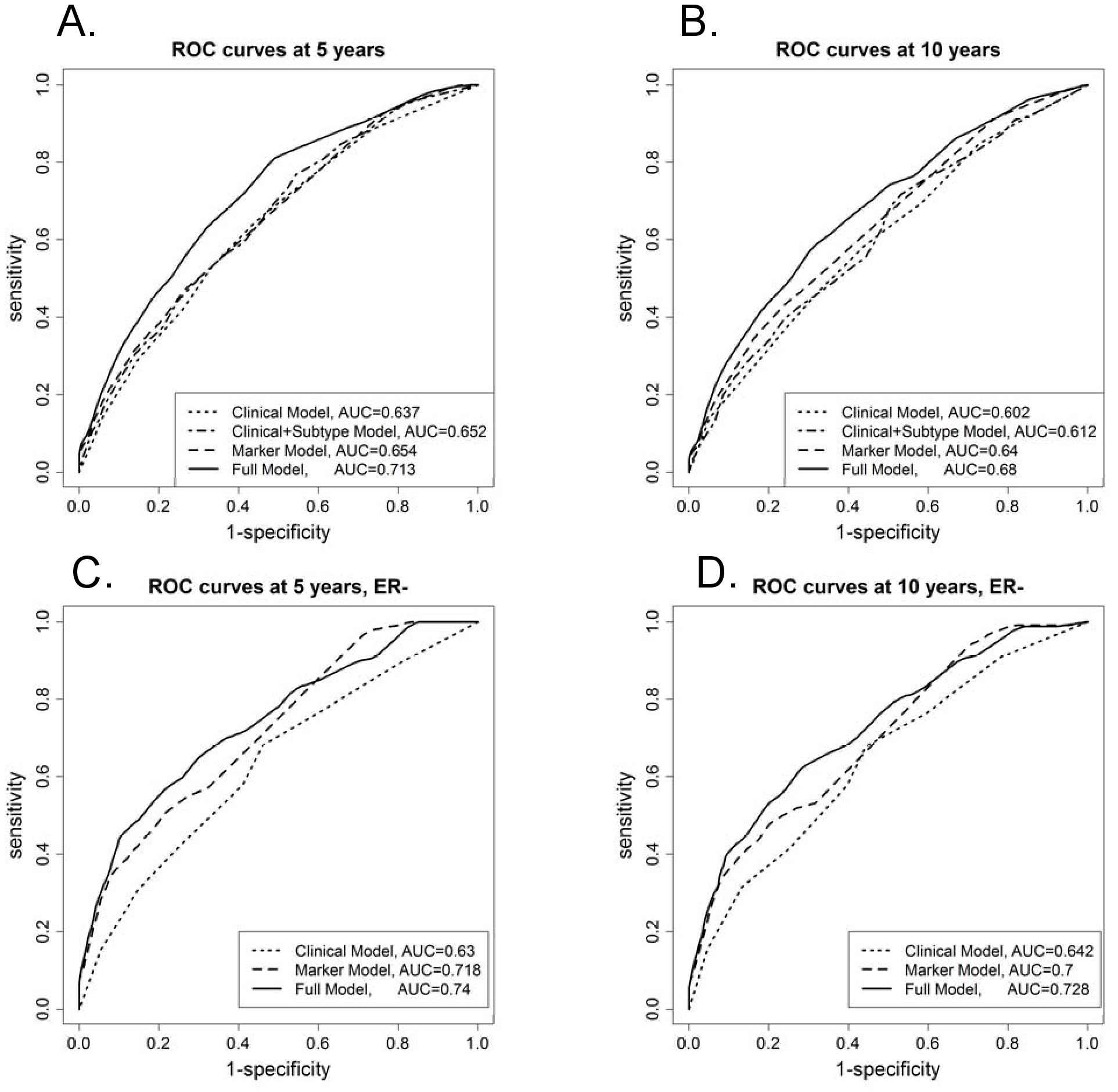

Supplement: Figure S3 — Time-dependent receiver operator characteristic (ROC) curves with the area under the curve (AUC) for the full models (19 CNIs, clinical, and tumor subtypes) compared to the clinical-only, clinical + tumor subtype, and 19-CNI ('marker only') models for 5-year (Panels A & C) and 10-year (Panels B & D) recurrence probability for all breast cancers (Panels A & B) and ER− cases only (Panels C & D). (TIFF) [file pone.0023543.s003.tiff]

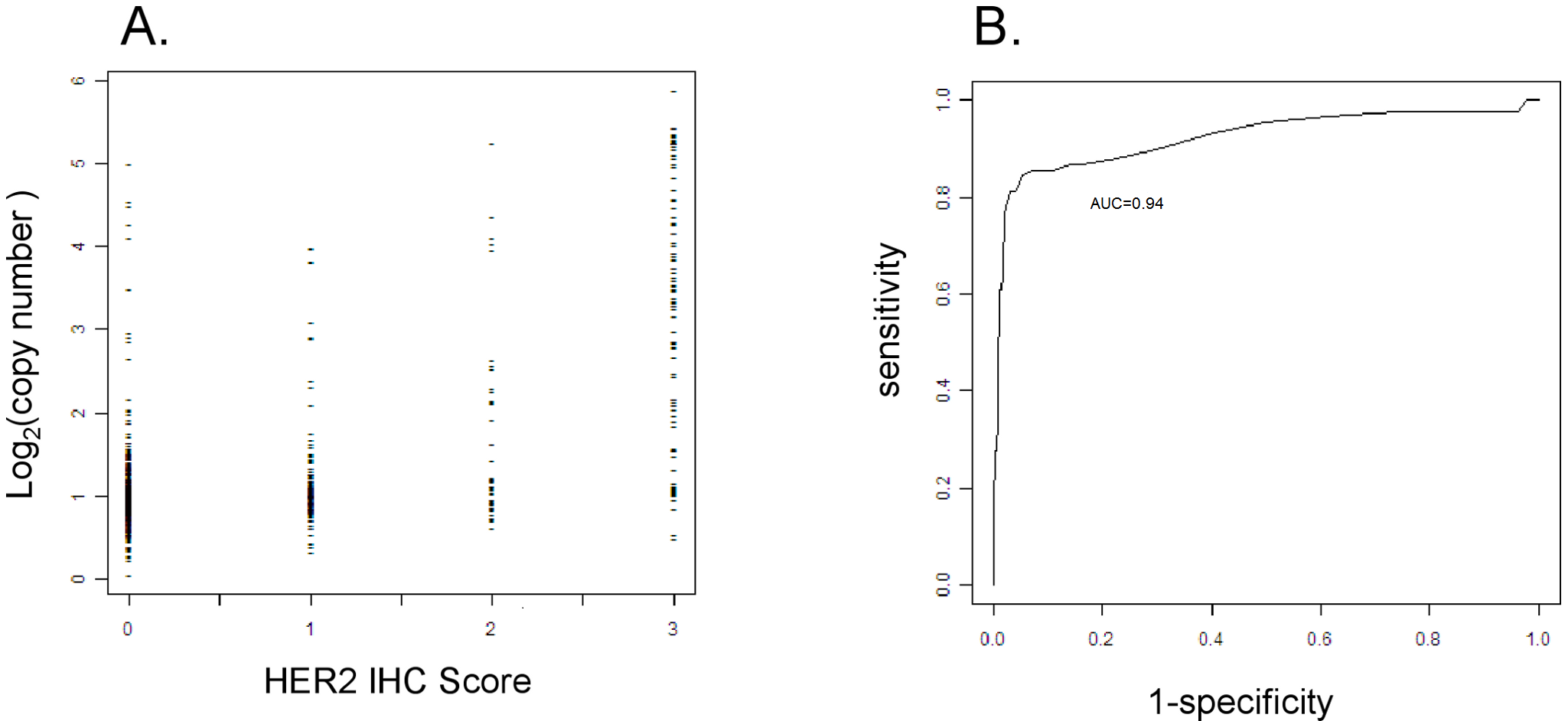

Supplement: Figure S4 — Determination of HER2 Status. (A) log2(copy number) by HER2 immunohistochemistry score from 848 breast tumors in tissue microarray studies. (B) Receiver Operator Curve (ROC) for the HER2 classifier based on copy number using a threshold of 2.8 as definition for gain. (TIFF) [file pone.0023543.s004.tiff]
