# Supplementary material for: Selective Genomic Copy Number Imbalances and Probability of Recurrence in Early-Stage Breast Cancer
Source: PLoS One. 2011 Aug 12;6(8):e23543. doi: 10.1371/journal.pone.0023543 (PMC3155554; doi:10.1371/journal.pone.0023543)
Supplement: Table S1 — Recurrent Copy Number Gains and Losses (≥10%) by Tumor Subtype. (DOCX) [file pone.0023543.s005.docx]

|  | Table S1. Recurrent Copy Number Gains and Losses ( ≥ 10%) by Tumor Subtype | | | | | | | |
| --- | --- | --- | --- | --- | --- | --- | --- | --- |
| Cytoband | | **Start-Stop** | **LUM A** | **LUM B** | **HER2+** | **TNBC** | **FDR** |  |
| 1p36.23-p36.31 | | nt6335810-nt7549455 |  | L (10.3%) |  |  | * |  |
| 1p12 | | nt119315210-nt132032120 |  |  |  | G (12%) | * |  |
| 1q | | nt132032121-nt246169833 | G (33.7%) | G (36.5%) | G (28.5) | G (29.9%) |  |  |
| 3p12.3-p12.1 | | nt75741512-nt85421052 |  |  |  | L (11.1%) | * |  |
| 3q22.3-q29 | | nt137439636-nt198436531 |  | G (12.2%) |  | G (17.4%) | * |  |
| 4p16.1-q35.2 | | nt9363894-nt190693052 |  |  |  | L (13%) | * |  |
| 4q13.3-q21.21 | | nt72460098-nt80491871 |  |  | G (11.1%) |  | * |  |
| 5p15.33-p13.1 | | nt513201-nt39107123 |  | G (10.2%) | G (10.8%) | G (10.9%) |  |  |
| 5q11.1-q35.1 | | nt47857293-nt169314864 |  |  |  | L (16.8%) | * |  |
| 6p25.3-p12.1 | | nt250828-nt57556632 |  |  |  | G (15.2%) | * |  |
| 6q16.2-q23.1 | | nt106591379-nt108103633 |  |  |  | G (11.4%) | * |  |
| 6q14.1-q27 | | nt79278474-nt170514911 |  | L (10.9%) |  |  | * |  |
| 7p21.3-p21.1 | | nt7323846-nt20541090 |  | G (10.6%) |  |  |  |  |
| 7q22.1-q35 | | nt97982815-nt143810122 |  |  |  | G (11.7%) | * |  |
| 8p23.3-p12 | | nt323049-nt37152648 | L (10%) | L (18.6%) | L (20.9%) | L (14.1%) |  |  |
| 8p11.23-q24.3 | | nt38759483-nt145205212 | G (15.9%) | G (31.4%) | G (33.5%) | G (31%) | * |  |
| 9p24.3-p21.3 | | nt296015-nt22639384 |  |  |  | G (17.1%) | * |  |
| 9p21.2-p21.1 | | nt22639385-nt30644743 |  | L (10.9%) | L (12%) | L (11.4%) | * |  |
| 10p15.3-p11.21 | | nt357955-nt39387588 |  |  | G (11.4%) | G (21.5%) | * |  |
| 11p13-p12 | | nt31571247-nt37948309 |  |  |  | G (13.1%) | * |  |
| 11q13.2-q13.3 | | nt68347506-nt70765491 | G (13.6%) | G (29.2%) | G (17.7%) | G (12.8%) | * |  |
| 11q14.1-q25 | | nt79277361-nt134112159 |  | L (17.3%) |  |  | * |  |
| 12p13.33-p11.21 | | nt141929-nt32858701 |  |  |  | G (15.8%) | * |  |
| 13q14.2-q31.1 | | nt47553393-nt86223773 |  | L (14.4%) | L (12.3%) | L (11.4%) |  |  |
| 13q33.3-q34 | | nt108039990-nt113501907 |  |  |  | G (12.5%) | * |  |
| 14q11.2 | | nt21452873-nt22182052 | G (56.3%) | G (44.9%) | G (32.3%) | G (21.7%) | * |  |
| 14q13.3-q32.31 | | nt36252347-nt101304688 |  |  |  | L (10.3%) | * |  |
| 15q12-q14 | | nt23182424-nt37936757 |  |  |  | L (12.2%) | * |  |
| 16p13.3-p13.13 | | nt239411-nt12100062 | G (20.8%) | G (16.7%) | G (10.1%) |  | * |  |
| 16p13.13-p11.2 | | nt12100063-nt35843070 | G (18.8%) | G (16.7%) |  |  | * |  |
| 16q12.1-q24.3 | | nt43075590-nt88440843 | L (19.8%) | L (19.9%) |  |  | * |  |
| 17p13.1 | | nt8360880-nt10577991 |  | L (16%) | L (13.3%) |  |  |  |
| 17p12 | | nt11878452-nt15865667 |  | L (14.4%) | L (13.9%) | L (10.3%) | * |  |
| 17q11.1-q23.2 | | nt22716824-nt57174968 |  |  | G (27.2%) |  | * |  |
| 17q23.2-q25.3 | | nt57174969-nt78241241 |  | G (11.9%) | G (26.3%) | G (17.4%) | * |  |
| 18p11.32-p11.21 | | nt342951-nt11479052 |  |  |  | G (10.9%) | * |  |
| 19q12-q13.2 | | nt28389533-nt45781867 |  |  | G (10.1%) | G (13.6%) | * |  |
| 20q13.13-q13.33 | | nt46969171-nt62114147 | G (11.6%) | G (20.5%) | G (27.8%) | G (10.9%) | * |  |
| 21q22.11-q22.3 | | nt34036402-nt46626193 |  |  |  | G (14.1%) | * |  |
| 22q11.1-q13.33 | | nt15236255-nt49264838 |  | L (12.2%) |  |  | * |  |
| Xp22.31-p11.23 | | nt2963446-nt49437557 |  |  |  | L (14.7%) | * |  |

* fdr <0.01
