# Supplementary material for: Selective Genomic Copy Number Imbalances and Probability of Recurrence in Early-Stage Breast Cancer
Source: PLoS One. 2011 Aug 12;6(8):e23543. doi: 10.1371/journal.pone.0023543 (PMC3155554; doi:10.1371/journal.pone.0023543)
Supplement: Table S2 — Expanded start and stop boundaries, segment size, and genes associated with 19 copy number imbalances selected for recurrence. (DOCX) [file pone.0023543.s006.docx]

| **Table S2.** Expanded start and stop boundaries, segment size, and genes associated with 19 copy number imbalances selected for recurrence. | | | |
| --- | --- | --- | --- |
| **Cytoband** | ***Start-Stop** | ***Length (kb)** | **Gene Symbol** |
| chr1p12 | nt119315210-nt119747280 | 432 | WARS2, HAO2, HSD3B2 |
| chr2p11.1 | nt91087616-nt94286916 | 3199 | GGT8P, ANKRD20B, MAL, MRPS5, TEKT4, |
| chr3q13.12,q13.13 | nt108059123-nt112251638 | 4193 | CCDC54, BBX, LOC151658, LOC285205, CD47, IFT57, HHLA2, KIAA1524, MYH15, DZIP3, RETNLB, TRAT1, GUCA1C, MORC1, DPPA2, DPPA4 |
| chr8p22 | nt16909831-nt18283695 | 1374 | EFHA2, ZDHHC2, CNOT7, VPS37A, MTMR7, PDGFRL, SLC7A2, MTUS1, FGL1, ASAH1, PCM1, NAT1 |
| chr10p13 | nt16084814-nt17528387 | 1444 | C1QL3,PTER,RSU1,CUBN,TRDMT1,VIM,ST8SIA6 |
| chr10p11.21 | nt36379031-nt37813659 | 1435 | ANKRD30A |
| chr10q22.3, q23.1 | nt81597709-nt82913296 | 1316 | ANXA11, C10orf57, PLAC9, C10orf58, DYDC1, DYDC2, MAT1A, TSPAN14, SH2D4B |
| chr11p15.1, p15.2 | nt14183576-nt19267810 | 5084 | RRAS2, COPB1, PSMA1, CALCA, CYP2R1, PDE3B, CALCB, INSC, SOX6, C11orf58, PIK3C2A, PLEKHA7, RPS13, KCNJ11, NUCB2, ABCC8, USH1C, KCNC1, MYOD1, SERGEF, MRGPRX3, SAA3P, SAAL1, TPH1, GTF2H1, HPS5, LOC494141, MRGPRX4, SAA1, SAA2, SAA4, LDHA , LDHAL6A, LDHC, TSG101, UEVLD, IGSF22, PTPN5, SPTY2D1, TMEM86A, MRGPRX1, MRGPRX2, CSRP3, E2F8, ZDHHC13 |
| chr11q13.5 | nt75235550-nt76296812 | 1061 | UVRAG, PRKRIR, WNT11, C11orf30, GUCY2E, LRRC32, TSKU |
| chr12p13.32, p13.33 | nt2195174-nt3630092 | 1435 | CACNA1C, FKBP4, ITFG2, NRIP2, C12orf32, FOXM1, TEAD4, TULP3, TSPAN9, PRMT8 |
| chr12q13.13 | nt50493755-nt51600159 | 1106 | ACVRL1, ANKRD33, ACVR1B, GRASP, C12orf44, NR4A1, KRT7, KRT80, KRT81, KRT83, KRT86, KRT6B, KRT75, KRT82, KRT84, KRT85, KRT5, KRT6A, KRT6C, KRT71, KRT72, KRT74, KRT1, KRT2, KRT73, KRT77, KRT3, KRT4, KRT76, KRT78, KRT79 |
| chr13q12.3 | nt28554115-nt29705278 | 1152 | KIAA0774, KIAA0774, SLC7A1, UBL3 |
| chr14q13.2,q13.3 | nt35380230-nt36252346 | 872 | MBIP, NKX2-1, SFTA3, NKX2-8, PAX9 |
| chr16p11.2 | nt31526202-nt35843070 | 4317 | C16orf58, C16orf67, ERAF, SLC5A2, TGFB1I1, ZNF267,Z NF720, HERC2P4, LOC440366, LOC729355, SLC6A10P, TP53TG3 |
| chr17q21.33 | nt47411130-nt48137311 | 726 |  |
| chr20q13.33 | nt59100794-nt60260005 | 1159 | CDH4, GTPBP5, LSM14B, PSMA7, SS18L1, TAF4 |
| chr22q11.1,q11.21 | nt15236255-nt16625906 | 1390 | CCT8L2, CECR8, XKR3, psiTPTE22, GAB4, CECR6, CECR7, IL17RA, CECR1, CECR4, CECR5, ATP6V1E1, CECR2, SLC25A18, BCL2L13, BID |
| chrXp21.1,p21.2 | nt30907133-nt33748113 | 2841 | DMD,DMD,DMD,DMD |
| chrXq28 | nt151081086-nt151871524 | 790 | GABRA3, MAGEA10, MAGEA5, CETN2, CSAG1, CSAG2, CSAG3, GABRQ, MAGEA12, MAGEA2, MAGEA2B, MAGEA3, MAGEA6, NSDHL, PNMA5, ZNF185 |

*Start and stop boundaries (and genes) are expanded to incorporate information on neighboring segments that are highly correlated (r^2^ >0.95) with the 19 CNIs.
