# Supplementary material for: Selective Genomic Copy Number Imbalances and Probability of Recurrence in Early-Stage Breast Cancer
Source: PLoS One. 2011 Aug 12;6(8):e23543. doi: 10.1371/journal.pone.0023543 (PMC3155554; doi:10.1371/journal.pone.0023543)
Supplement: Table S3 — Frequency of 19 Copy Number Imbalances by Subtype. (DOCX) [file pone.0023543.s007.docx]

| **Table S3. Frequency of 19 Copy Number Imbalances by Subtype** | | | | | | | | | | | | |
| --- | --- | --- | --- | --- | --- | --- | --- | --- | --- | --- | --- | --- |
| ***Cytoband no. (%)** | ****Subtypes** | | | | | | | | | | | |
|  | LUM A (N=389) | | | LUM B (N=156) | | | HER2+ (n=158) | | | TNBC (N=184) | | |
|  | Loss | Normal | Gain | Loss | Normal | Gain | Loss | Normal | Gain | Loss | Normal | Gain |
| 1p12 | 8 (2.1) | 374 (96.1) | 7 (1.8) | 4 (2.6) | 138 (88.5) | 14 (9.0) | 2 (1.3) | 148 (93.7) | 8 (5.1) | 1 (0.5) | 163 (88.6) | 20 (10.9) |
| 2p11.1 | 2 (0.5) | 385 (98.0) | 2 (0.5) | 0 (0) | 153 (98.1) | 3 (2.0) | 0 (0) | 158 (100) | 0 (0) | 1 (0.5) | 170 (92.4) | 13 (7.1) |
| 3q13.12 | 3 (0.8) | 381 (98.0) | 5 (1.3) | 0 (0) | 149 (95.5) | 7 (4.5) | 2 (1.3) | 149 (94.3) | 7 (4.4) | 5 (2.7) | 172 (93.5) | 7 (3.8) |
| 8p22 | 39 (10.0) | 333 (85.6) | 17 (4.4) | 28 (18.0) | 116 (74.4) | 12 (7.7) | 32 (20.3) | 117 (74.1) | 9 (5.7) | 22 (12.0) | 156 (84.8) | 6 (3.3) |
| 10p13 | 3 (0.8) | 378 (97.2) | 8 (2.1) | 2 (1.3) | 142 (91.0) | 12 (7.7) | 1 (0.6) | 137 (86.7) | 20 (12.7) | 0 (0) | 147 (79.9) | 37 (20.1) |
| 10p11.21 | 3 (0.8) | 378 (97.2) | 8 (2.1) | 0 (0) | 149 (95.5) | 7 (4.5) | 1 (0.6) | 147 (93.0) | 10 (6.3) | 0 (0) | 158 (85.9) | 26 (14.1) |
| 10q23.1 | 8 (2.1) | 372 (95.6) | 9 (2.3) | 2 (1.3) | 149 (95.5) | 5 (3.2) | 2 (1.3) | 150 (95.0) | 6 (3.8) | 4 (2.2) | 170 (92.4) | 10 (5.4) |
| 11p15 .1 | 4 (1.0) | 377 (96.9) | 8 (2.1) | 5 (3.2) | 146 (93.6) | 5 (3.2) | 4 (2.5) | 154 (97.5) | 0 (0) | 6 (3.3) | 167 (90.8) | 11 (6.0) |
| 11q13.5 | 10 (2.6) | 350 (90.0) | 29 (7.5) | 8 (5.1) | 125 (80.1) | 23 (14.7) | 4 (2.5) | 145 (91.87) | 9 (5.7) | 4 (2.2) | 166 (90.2) | 14 (7.6) |
| 12p13.3 | 5 (1.3) | 376 (96.7) | 8 (2.1) | 1 (0.6) | 142 (91.0) | 13 (8.3) | 0 (0) | 150 (95.0) | 8 (5.1) | 4 (2.2) | 144 (78.3) | 36 (19.6) |
| 12q13.1 | 1 (0.3) | 381 (98.0) | 7 (1.1) | 2 (1.3) | 145 (93.0) | 9 (5.8) | 0 (0) | 148 (93.7) | 10 (6.3) | 11 (6.0) | 170 (92.4) | 3 (1.6) |
| 13q12.3 | 17 (4.4) | 368 (94.6) | 4 (1.0) | 14 (9.0) | 134 (86.0) | 8 (5.1) | 14 (8.9) | 134 (84.8) | 10 (6.3) | 11 (6.0) | 169 (91.9) | 4 (2.2) |
| 14q13.2 | 2 (0.5) | 371 (95.4) | 16 (4.1) | 4 (2.6) | 143 (91. 7) | 9 (5.8) | 6 (3.8) | 139 (88.0) | 13 (8.2) | 17 (9.2) | 162 (88.0) | 5 (2.7) |
| 16p11.2 | 3 (0.8) | 327 (84.1) | 59 (15.2) | 4 (2.6) | 130 (83. 3) | 22 (14.1) | 0 (0) | 140 (88.6) | 18 (11.4) | 1 (0.5) | 172 (93.5) | 11 (6.0) |
| 17q21.33 | 5 (1.3) | 365 (93.8) | 19 (4.9) | 1 (0.6) | 135 (86.5) | 20 (12.8) | 1 (0.6) | 119 (75.3) | 38 (24.1) | 5 (2.7) | 172 (93.5) | 7 (3.8) |
| 20q13.33 | 2 (0.5) | 342 (87.9) | 45 (11.6) | 1 (0.6) | 125 (80.1) | 30 (19.2) | 0 (0) | 112 (70.9) | 46 (29.1) | 3 (1.6) | 163 (88.6) | 18 (9.8) |
| 22q11.1 | 24 (6.2) | 361 (92.8) | 4 (1.0) | 16 (10.3) | 136 (87.2) | 4 (2.6) | 7 (4.4) | 143 (90.5) | 8 (5.1) | 3 (1.6) | 170 (92.4) | 11 (6.0) |
| Xp21.1 | 10 (2.6) | 372 (95.6) | 7 (1.8) | 7 (4.5) | 146 (93.6) | 3 (1.9) | 13 (8.2) | 137 (86.7) | 8 (5.1) | 30 (16.3) | 150 (81.5) | 4 (2.2) |
| Xq28 | 13 (3.3) | 371 (95.4) | 5 (1.3) | 8 (5.1) | 145 (93.0) | 3 (1.9) | 9 (5.7) | 139 (88.0) | 10 (6.3) | 7 (3.8) | 166 (90.2) | 11 (6.0) |

*For detailed annotation of chromosomal segments see Supplemental Table 2.

*84 patients with missing subtypes information.
