# Supplementary material for: Selective Genomic Copy Number Imbalances and Probability of Recurrence in Early-Stage Breast Cancer
Source: PLoS One. 2011 Aug 12;6(8):e23543. doi: 10.1371/journal.pone.0023543 (PMC3155554; doi:10.1371/journal.pone.0023543)
Supplement: Table S4 — Posterior Probabilities for the individual 19 CNIS for the Full and 19 CNI Only Models. (DOCX) [file pone.0023543.s008.docx]

| **Table S4. Posterior Probabilities for the individual 19 CNIs for the Full and 19 CNI Only Models.** | | | | | | | | |
| --- | --- | --- | --- | --- | --- | --- | --- | --- |
|  |  | ***Full Model** | | | **19 CNI Only model** | | | |
| **Cytoband*** | **Start-Stop** | **Posterior Probability**  **(%)** | **Hazard**  **Ratio** | **p-value** | | **Posterior Probability**  **(%)** | **Hazard**  **Ratio** | **p-value** |
| 10p13 | nt16084814-nt17528387 | 100 | 2.11 | <0.000 | | 100 | 2.09 | <0.000 |
| 11q13.5 | nt75779338-nt76296812 | 100 | 1.64 | 0.003 | | 100 | 1.62 | 0.004 |
| 22q11.1,q11.21 | nt15236255-nt16625906 | 100 | 0.43 | <0.000 | | 100 | 0.44 | <0.000 |
| Xp21.1,p21.2 | nt30907133-nt32653344 | 100 | 0.50 | <0.000 | | 100 | 0.46 | <0.000 |
| 3q13.12,q13.13 | nt108059123-nt112251638 | 99.9 | 2.95 | <0.000 | | 99.8 | 2.72 | <0.000 |
| Xq28 | nt151081086-nt151871524 | 99.8 | 1.93 | 0.001 | | 98.4 | 1.90 | 0.001 |
| 14q13.2,q13.3 | nt35380230-nt36252346 | 97 | 1.77 | 0.004 | | 98.4 | 1.81 | 0.003 |
| 8p22 | nt17229368-nt17457649 | 80.9 | 0.67 | 0.004 | | 68.4 | 0.68 | 0.005 |
| 17q21.33 | nt 47411130-nt48137311 | 56.3 | 0.61 | 0.012 | | 67.1 | 0.61 | 0.011 |
| 10p11.21 | nt36379031-nt37813659 | 48 | 0.46 | 0.009 | | 59.7 | 0.46 | 0.007 |
| 12p13.32 | nt3394093-nt3630092 | 40 | 1.75 | 0.004 | | 34 | 1.67 | 0.007 |
| 12q13.13 | nt50493755-nt51600159 | 36.4 | 0.58 | 0.036 | | 21.1 | 0.63 | 0.065 |
| 10q23.1 | nt82273705-nt82913296 | 26.8 | 1.84 | 0.007 | | 62.6 | 1.96 | 0.002 |
| 1p12 | nt119315210-nt119747280 | 20.3 | 0.54 | 0.011 | | 33.5 | 0.54 | 0.008 |
| 2p11.1 | nt91087616-nt94286916 | 11.7 | 1.96 | 0.053 | | 9.9 | 1.89 | 0.065 |
| 13q12.3 | nt28554115-nt29380652 | 5.4 | 1.46 | 0.053 | | 7.8 | 1.46 | 0.053 |
| 16p11.2 | nt31526202-nt35843070 | 5 | 0.74 | 0.111 | | 29.8 | 0.69 | 0.033 |
| 20q13.33 | nt59456751-nt59788832 | 3.4 | 1.34 | 0.065 | | 21.7 | 1.40 | 0.026 |
| 11p15.1, p15.2 | nt14183576-nt19267810 | 0.6 | 1.32 | 0.316 | | 0.7 | 1.28 | 0.342 |

*Full model (Clinical covariates, tumor subtypes, and 19 CNIs)

**Cytobands are shown in order relative to their posterior probability
